# The association between serum albumin and depressive symptoms: a cross-sectional study of NHANES data during 2005–2018

**DOI:** 10.1186/s12888-023-04935-1

**Published:** 2023-06-20

**Authors:** Guimei Zhang, Shuna Li, Sisi Wang, Fangyi Deng, Xizhe Sun, Jiyang Pan

**Affiliations:** 1 Department of Psychiatry, Sleep Medicine Centre, First Affiliated Hospital of Jinan University, Guangzhou, 510632 Guangdong Province P.R. China; 2 Department of Clinical Research, The First Affiliated Hospital of Jinan University, Guangzhou, 510632 Guangdong P.R. China

**Keywords:** Serum albumin, Depressive symptoms, NHANES, Adults

## Abstract

**Aims:**

The association between serum albumin and depressive symptoms has been unclear in previous epidemiological studies. We explored whether serum albumin is associated with depressive symptoms based on the National Health and Nutrition Examination Survey (NHANES) data.

**Methods:**

This cross-sectional study included 13,681 participants aged ≥ 20 years from the NHANES performed during 2005–2018, which produced nationally representative database. Depressive symptoms were assessed using the Patient Health Questionnaire-9. Serum albumin concentration was measured using the bromocresol purple dye method, and participants were divided into quartiles of serum albumin concentrations. Weighted data were calculated according to analytical guidelines. Logistics regression and linear regression models were used to assess and quantify the association between serum albumin and depressive symptoms. Univariate and stratified analyses were also performed.

**Results:**

There were 1551 (10.23%) adults (aged ≥ 20 years) with depressive symptoms among the 13,681. A negative association was found between serum albumin concentration and depressive symptoms. Compared with the lowest albumin quartile, the multivariate-adjusted effect size (95% confidence interval) for depressive symptoms of the fully adjusted model in the highest albumin quartile was 0.77 (0.60 to 0.99) and − 0.38 (− 0.66 to − 0.09) using logistics regression and linear regression models respectively. Current smoking status modified the association between serum albumin concentration and PHQ-9 scores (p for interaction = 0.033).

**Conclusion:**

This cross-sectional study revealed that albumin concentration is significantly more likely to be a protective factor for depressive symptoms, with the association being more pronounced in non-smokers.

**Supplementary Information:**

The online version contains supplementary material available at 10.1186/s12888-023-04935-1.

## Introduction

Depressive disorder is one of the leading global health issues [1], and is clinically characterized by significant and persistent low mood symptoms, and impairments in cognition, emotional regulation, memory, motor function, motivation, and neurovegetative symptoms [2]. The World Health Organization estimated that depression will account for 13% of global diseases by 2030 and replace cardiovascular disease as the disease with the greatest burden [3]. Depression can lead to various functional physical impairments and loss of interest in daily activities, thereby reducing the quality of life [4]. It can cause huge detriments to health correlated with chronic medical illnesses such as cardiovascular disease [5], diabetes [6], and cancer [7]. Furthermore, depression has been reported to be the most common cause of suicide, and it is currently one of the top-ten most common causes of death in the US. The combination of the primary disability caused by depression and the secondary disability of chronic medical illness results in depression being one of the costliest medical burdens in the world. The number of adults in the US with depressive disorder increased by 12.9% between 2010 and 2018, from 15.5 to 17.5 million. Over this period, the incremental economic burden of adults with depressive disorder increased by 37.9% from $USD 236.6 billion to $USD 326.2 billion (based on 2020 values). All components of the incremental economic burden increased (i.e., direct, suicide-related, and workplace costs), with the largest increase of 73.2% observed in workplace costs [8]. Depression onset therefore needs to receive urgent attention.

Albumin is the most abundant protein in human plasma and has various essential functions, including being an important carrier protein for various steroids, hormones, and fatty acids, drug binding, antioxidant activity, inflammatory response, and immune regulation [9]. Furthermore, osmotic blood pressure maintenance could not occur without plasma albumin [10]. Serum albumin levels are also clinically useful in assessing liver function, kidney function, and nutritional status [11]. Albumin has been identified as an important nonenzymatic antioxidant [12].

Albumin levels gradually increase after antidepressant treatment [13], and lower albumin levels in patients in remission from depressive disorder may increase the risk of depressive relapse [14]. In Western countries, low albumin levels or hypoalbuminemia coexist as drug resistance develops in patients with depression [15]. These findings suggest that serum albumin is involved in the pathophysiological processes of depression. However, the direct association between depressive symptoms and serum albumin is currently unclear.

This cross-sectional study tested the hypothesis that albumin affects depression. We conducted this study using data from the National Health and Nutrition Examination Survey (NHANES) performed during 2005–2018 to investigate the correlation between depressive symptoms and albumin concentrations in adults.

## Methods

### Study design

The NHANES is a US national stratified multistage probability sampling program performed by the National Center for Health Statistics (NCHS) to assess nutritional status and its associations with health promotion and disease prevention. The survey comprises a combination of interviews and physical examinations. Highly trained medical personnel administer the examinations and laboratory tests.

**Fig. 1:**
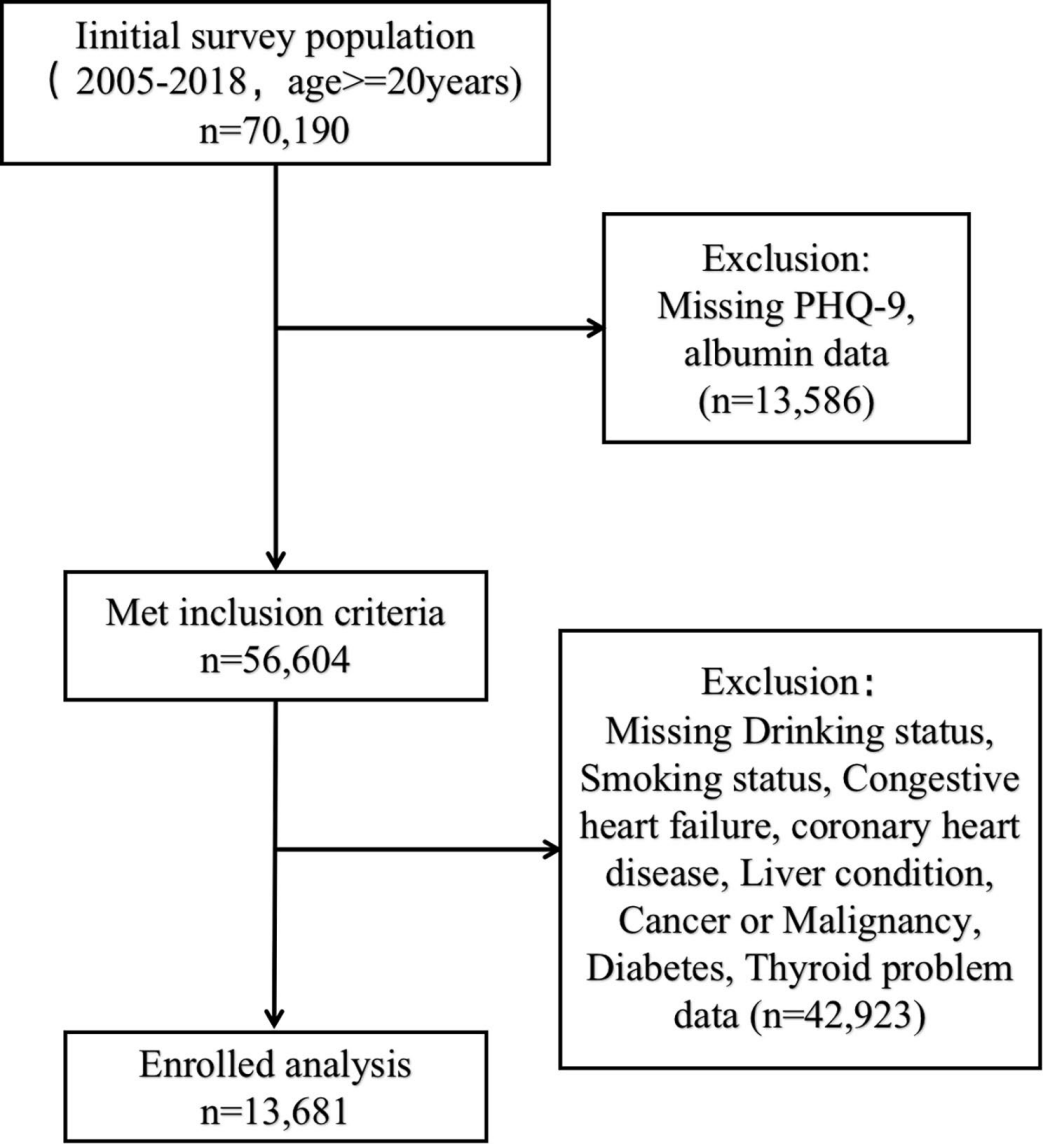
Flow chart of participants included

This study, which used data from the NHANES performed during 2005–2018, was approved by the NCHS Research Ethics Review Board, and written consent was obtained from all surveyed individuals by the Centers for Disease Control and Prevention. The rigorous screening of 70,190 participants identified 13,681 participants aged ≥ 20 years with a complete set of Patient Health Questionnaire-9 (PHQ-9), albumin, and covariate data, who were included in this study. The detailed screening process of participants is shown in Fig. 1.

### Data collection and definitions

#### Depressive symptoms

Depressive symptoms was assessed using PHQ-9 [16], which is a self-reported depression screening tool based on the nine items that reflect depressive disorder in the Fifth Edition of the Diagnostic and Statistical Manual of Mental Disorders. PHQ-9 is a reliable and valid measure of depression severity that asks questions about the frequency of depressive symptoms experienced over the past 2 weeks. Each question is scored from 0 (not at all) to 3 (nearly every day), with the final questionnaire score ranging from 0 to 27. Scores of at least 10 were considered to indicate depressive symptoms [17].

#### Albumin

Albumin concentration was measured using the bromocresol purple dye method. Serum specimens are processed, stored under appropriate refrigerated (2–8 °C) conditions, and shipped to Collaborative Laboratory Services for testing and analysis. The DcX800 method is used to measure the albumin concentration as a bichromatic digital endpoint method. In the reaction, the albumin combines with Bromcresol Purple (BCP) reagent to form a complex. The absorbance was tested at 600 nm and monitored with the concentration of albumin. The change in absorbance is directly proportional to the concentration of albumin in the sample. The NHANES Quality Control and Quality Assurance Protocol complies with the requirements of the Clinical Laboratory Improvement Amendments of 1988.

#### Covariates

Based on the existing literature [18], the following variables that were potential confounders in the association between albumin and depressive symptoms were assessed: gender, age, race, education level, smoking status, and drinking status. Current smoking status was categorized into smoking and nonsmoking by the question “Do you smoke now?” Drinking status was categorized into drinking and nondrinking by the question “Do you drink alcohol now?” Body mass index (BMI) was calculated by trained health technicians at the Mobile Examination Center (MEC) as the weight in kilograms divided by the height in meters squared. Chronic diseases were assessed by the self-reported medical history, including that for congestive heart failure, coronary heart disease, liver function, cancer or malignancy, diabetes, and thyroid problems. The assessment involved asking whether or not the doctor had informed the participant if they had any of these conditions.

### Statistical analysis

All the interviews and MEC examination weights covered in this study are available in the demographic files. The detailed data are available at the following website: https://wwwn.cdc.gov/nchs/nhanes/tutorials/module3.aspx. Since those examined by the MEC were a subset of those interviewed in the survey, we combined the MEC examination weights for the analysis. The NHANES performed during 2005–2018 involved seven survey cycles spanning 14 years, and the data were weighted according to the information that the NCHS analysts provided on how to combine multiple cycles and construct appropriate weights. Multivariate logistic regression models were used to describe the association between albumin concentration and depressive symptoms. We constructed three models respectively: (1) model I included no adjustment; (2) model II adjusted for age, gender, and race; and (3) model III adjusted for age, race, gender, education level, BMI, drinking status, smoking status, congestive heart failure, coronary heart disease, liver function, cancer or malignancy, diabetes, and thyroid problems. We also used linear regression models to describe the association between albumin concentration and PHQ-9 scores from the three similar models as above and decreased the skewness distribution of PHQ-9 scores (Square root transformation). Stratified analyses with subgroup variables were performed using the fully adjusted model. We performed univariate and stratified analyses to identify independent effects between PHQ-9 scores and albumin concentration. Chi-squared or Kruskal-Wallis H tests were applied to different albumin quartile groups. An interaction test was also performed on this factor. Continuous variables were represented by mean ± standard deviation values, while percentages were used to represent classified variables. The effect value was expressed as odds ratios (OR), β and corresponding 95% confidence interval (CI). All analyses were performed using the statistical software R (version 3.6.1, https://www.r-project.org/). A two-sided probability value of p < 0.05 was considered significant in all analyses.

## Results

This study included 13,681 adults aged 48.97 ± 0.27 years, including 56.74% males and 74.28% non-Hispanic whites. There were 1551 (10.23%) participants with depressive symptoms. Weighted baseline characteristics according to albumin quartiles are listed in Table 1. Those with higher albumin concentrations were more likely to be male, have a higher education level, and be nonsmokers and nondrinkers. The proportion of people with disease (diabetes, thyroid problems, coronary heart disease, liver function, congestive heart failure, cancer/malignancy, and depressive symptoms) was higher among those in the highest albumin quartile (Q4) than in the lowest albumin quartile. The albumin quartile was significantly associated with age, BMI, gender, race, education level, smoking status, diabetes, thyroid problems, coronary heart disease, liver function, congestive heart failure, cancer or malignancy, and depressive symptoms (all p < 0.001), but not drinking status (p = 0.385).

**Table 1:** Weighted baseline characteristics according to albumin quartile

Albumin quartile		Q1	Q2	Q3	Q4	Total	*p*
N		2745.00	2744.00	3292.00	4900.00	13681.00	
Age(years) $\tiny \left( {\overline X \pm SD} \right)$		52.45 ±16.50	50.97 ±16.27	49.55 ±15.88	43.82 ± 16.00	48.97 ± 0.27	< 0.001
BMI status $\tiny \left( {\overline X \pm SD} \right)$		31.91 ± 8.17)	29.38 ± 6.52	28.39 ± 5.83	26.71 ± 4.93	29.02 ± 0.09	< 0.001
Gender (%)	Male	1831 (40.67)	1788 (51.60)	2084 (61.17)	2523 (71.22)	8226 (56.74)	< 0.001
	Female	2140 (59.33)	1371 (48.40)	1083 (38.83)	861 (28.78)	5455 (43.26)	
Race (%)	Mexican American	460 (6.45)	425 (6.67)	443 (6.66)	450 (6.65)	1778 (6.61)	< 0.001
	Other Hispanic	297 (4.12)	260 (4.43)	267 (4.27)	286 (4.30)	1110 (4.28)	
	Non-Hispanic White	1905 (69.88)	1591 (73.45)	1691 (76.21)	1863 (77.17)	7050 (74.28)	
	Non-Hispanic Black	1066 (13.91)	670 (9.98)	506 (7.08)	476 (5.84)	2718 (9.09)	
	Other Race	243 (5.64)	213 (5.47)	260 (5.79)	309 (6.05)	1025 (5.75)	
Education level (%)	Less than 9th grade	376 (5.41)	290 (4.94)	296 (4.59)	275 (4.44)	1237 (4.83)	< 0.001
	9-11th grade	769 (16.02)	548 (13.27)	504 (12.49)	548 (11.88)	2369 (13.37)	
	High school graduate/GED or equivalent	1061 (30.15)	820 (26.44)	839 (27.00)	896 (26.18)	3616 (27.43)	
	Some college or AA degree	1253 (32.37)	996 (35.32)	947 (32.99)	1009 (32.39)	4205 (33.19)	
	College graduate or above	512 (16.05)	505 (20.04)	581 (22.93)	656 (25.11)	2254 (21.18)	
Smoking status (%)	Yes	1797 (44.72)	1450 (44.53)	1411 (43.86)	1605 (44.90)	6263 (44.52)	< 0.001
	No	2174 (55.28)	1709 (55.47)	1756 (56.14)	1779 (55.10)	7418 (55.48)	
Drinking status (%)	Yes	1022 (24.66)	785 (23.81)	866 (26.15)	915 (26.08)	3588 (25.23)	0.358
	No	2949 (75.34)	2374 (76.19)	2301 (73.85)	2469 (73.92)	10,093 (74.77)	
Diabetes (%)	Yes	778 (16.40)	437 (10.56)	363 (8.76)	303(6.05)	1881 (10.31)	< 0.001
	No	3084 (81.11)	2637 (87.21)	2716 (88.98)	3007 (91.80)	11,444 (87.42)	
	Borderline	109 (2.49)	85(2.23)	88(2.25)	74 (2.15)	356 (2.28)	
Thyroid problem (%)	Yes	517 (14.59)	370 (13.61)	268 (9.27)	213 (7.04)	1368 (10.94)	< 0.001
	No	3454 (85.41)	2789 (86.39)	2899 (90.73)	3171 (92.96)	12,313 (89.06)	
Coronary heart disease (%)	Yes	296 (6.81)	190 (5.04)	159 (4.53)	117 (2.97)	762 (4.77)	< 0.001
	No	3675 (93.19)	2969 (94.96)	3008 (95.47)	3267 (97.03)	12,919 (95.23)	
Liver condition (%)	Yes	263 (5.92)	159 (5.14)	141 (3.90)	144 (3.71)	707 (4.63)	< 0.001
	No	3708 (94.08)	3000 (94.86)	3026 (96.10)	3240 (96.29)	12,974 (95.37)	
Congestive heart failure (%)	Yes	283 (5.86)	128 (3.05)	85 (2.23)	67 (1.27)	563 (3.05)	< 0.001
	No	3688 (94.14)	3031 (96.95)	3082 (97.77)	3317 (98.73)	13,118 (96.95)	
Cancer or Malignancy (%)	Yes	558 (14.37)	369 (12.67)	357 (12.46)	257 (8.48)	1541 (11.86)	< 0.001
	No	3413 (85.63)	2790 (87.33)	2810 (87.54)	3127 (91.52)	12,140 (88.14)	
Depression (%)	Yes	577 (14.01)	369 (10.21)	316 (9.12)	289 (7.85)	1551 (10.23)	< 0.001
	No	3394 (85.99)	2790 (89.79)	2851 (90.88)	3095 (92.15)	12,130 (89.77)	

**Fig. 2:**
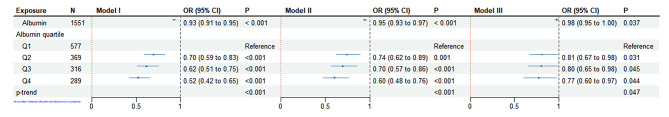
Weighted association between albumin and depressive symptoms based on logistics regression models Data are presented as odds ratio (OR), 95% confidence intervals, and *p*-value Model I adjust for: none Model II adjust for: age (years), gender, race Model III adjust for: age (years), race, gender, education level, BMI status, drinking status, smoking status, congestive heart failure, coronary heart disease, liver condition, cancer or malignancy, diabetes, thyroid problem

Figure 2 presents the association between albumin concentration and depression symptoms in logistic regression models. Overall, compared with quartile 1, individuals with higher albumin concentration had lower odds of depressive symptoms by 30% (OR = 0.70, 95% CI = 0.59–0.83), 38% (OR = 0.62, 95% CI = 0.51–0.75) and 48% (OR = 0.52, 95% CI = 0.42–0.65) respectively (after adjusting for none). After adjusting for age, gender, and race, with increasing quartiles of albumin levels, the corresponding OR (95%CI) were 0.74 (95% CI = 0.62–0.89) for quartile 2, 0.70 (0.57 to 0.86) for quartile 3, and 0.60 (0.48 to 0.76) for quartile 4, respectively (p trend < 0.001). When further adjusted for education level, BMI status, drinking status, smoking status, congestive heart failure, coronary heart disease, liver function, cancer or malignancy, diabetes, and thyroid problems, the association still persisted. For example, in comparison with the lowest albumin quartile, people with second albumin quartile were associated with a 19% (OR = 0.81, 95% CI = 0.67–0.98) lower odds of depressive symptoms, and lower odds of depressive symptoms were also found in people with third albumin quartile (OR = 0.80, 95% CI = 0.65–0.98) and highest albumin quartile (OR = 0.77, 95% CI = 0.60–0.97) significantly (p trend < 0.001). When albumin was analyzed as a continuous variable, participants with higher albumin concentration was significantly associated with lower odds of depressive symptoms by 5% (OR = 0.95, 95% CI = 0.93–0.97) and 2% (OR = 0.98, 95% CI = 0.95–0.99) respectively, (after adjusting for age, gender, and race and all factors), relative to their counterparts without depressive symptoms.

**Fig. 3:**
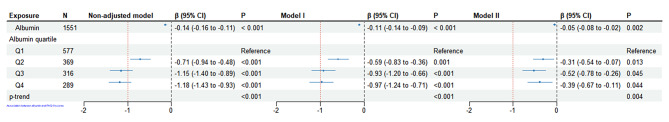
Weighted association between albumin and PHQ-9 scores in linear regression models Data are presented as β, 95% confidence intervals, and *p*-value Non-adjusted model adjusts for: none Adjust I model adjust for: age (years), gender, race Adjust II model adjust for: age (years), race, gender, education level, BMI status, drinking status, smoking status, congestive heart failure, coronary heart disease, liver condition, cancer or malignancy, diabetes, thyroid problem

Figure 3 presents the association between albumin concentration and PHQ-9 scores based on three linear regression models. When albumin was used as a continuous variable, a negative association was found between albumin concentration and PHQ-9 scores (β = −0.14, 95% CI = − 0.16 to − 0.11) in the non-adjusted model. After adjusting for age, gender, and race, the negative association was still present (β = −0.11, 95% CI = − 0.14 to − 0.09). The association weakened (but still persisted) after further adjusting for education level, BMI status, drinking status, smoking status, congestive heart failure, coronary heart disease, liver function, cancer or malignancy, diabetes, and thyroid problems (β = −0.05, p = 0.002). There was still an inverse association between albumin quartile and PHQ-9 scores in the three models. Compared with the lowest albumin quartile, the multivariate-adjusted coefficients and their 95% CIs for depressive symptoms in adjusted model II were − 0.31 (− 0.14 to − 0.09) for quartile 2, − 0.52 (− 0.78 to − 0.26) for quartile 3, and − 0.38 (− 0.66 to − 0.09) for quartile 4, after decreasing the skewness distribution of PHQ-9 scores by square root transformation, the association also still persisted significantly (p < 0.05) (seen supplemented Fig. 1).

**Fig. 4:**
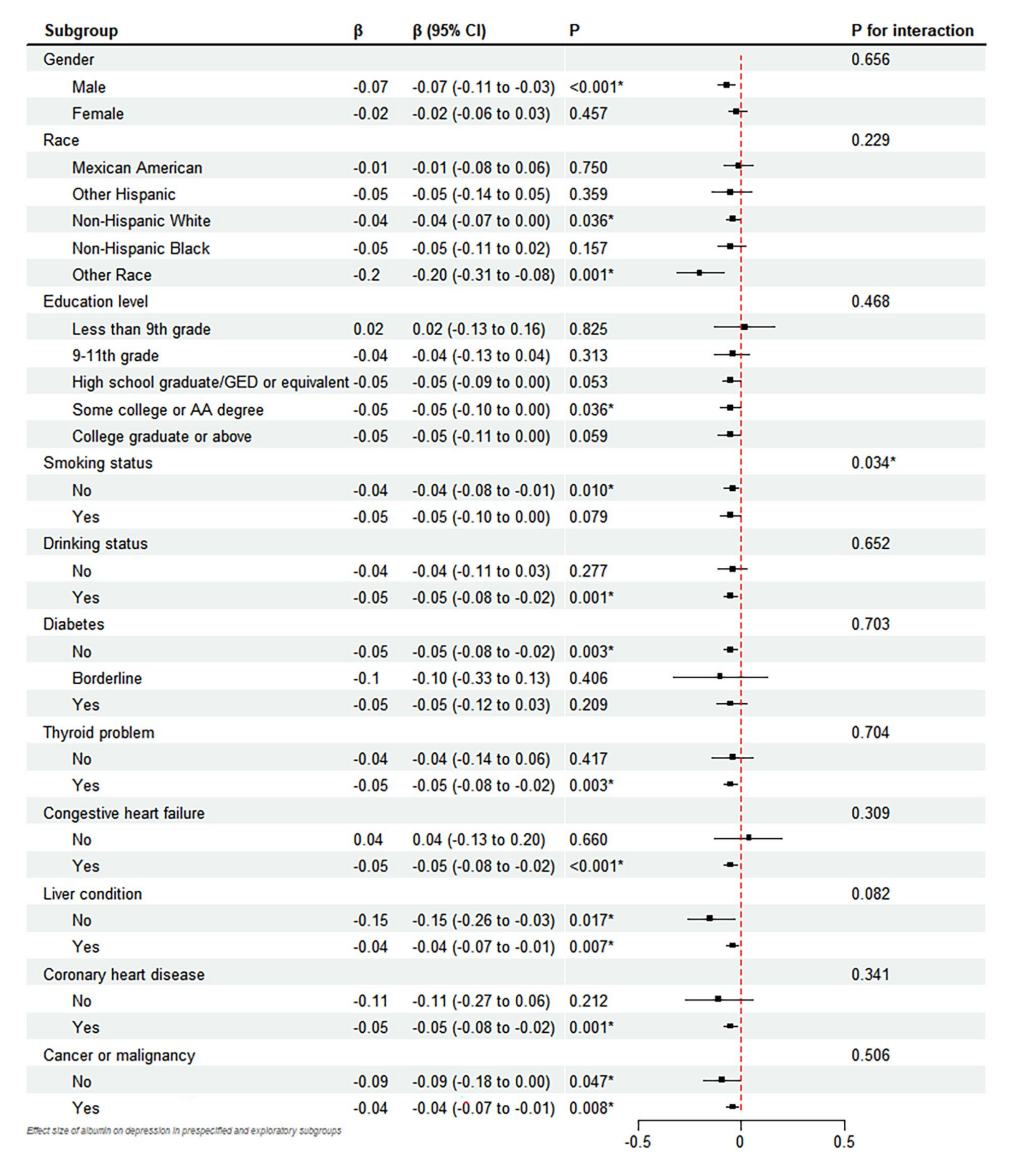
The forest plot shows the weighted effect size (β) of albumin on PHQ-9 scores in prespecified and exploratory subgroups based on linear regression Each stratification adjusted for all factors (age, race, gender, education level, BMI status, drinking status, smoking status, congestive heart failure, coronary heart disease, liver condition, cancer or malignancy, diabetes, thyroid problem) except the stratification factor itself. *: *p* < 0.05

Subgroup analysis was performed to estimate the robustness of the association between albumin concentration and depressive symptoms. In adjusted model II, the association between albumin and depressive symptoms was similar in most subpopulations (all p for interaction > 0.05), except when stratified by smoking status (p = 0.033) (Fig. 4), and the interaction analysis was performed for age (β = -0.0005, 95% CI= -0.002 to 0.001) and BMI (β = -0.001 ,95% CI = -0.05 to 0.02, P = 0.378), there was no significance statistically.

## Discussion

The main purpose of this national representative cross-sectional study was to determine the association between albumin and depressive symptoms. The results indicated that serum albumin level was negatively associated with depressive symptoms in adults when it was included as a continuous or an interquartile variable. Meanwhile, the association between albumin and the depressive symptoms was still consistent in various subgroup analyses, except when stratified by smoking status.

Our findings for the association between albumin and depressive symptoms were similar to previous reports [19–23], but those were mostly related to comorbid physical diseases and hence did not represent the direct association between albumin and depression. Albumin could therefore be a novel indicator for evaluating the progress and severity of depression [24]. Albumin levels could affect the development of depression since serum albumin levels are significantly lower in patients with depressive disorder than in healthy individuals [25, 26] and are correlated with disease severity [24, 27]. Albumin levels also gradually increase following antidepressant treatment [13]. Reduced albumin levels in patients in remission from depressive disorder may increase the risk of depressive relapse [14]. In Western countries, low albumin levels (i.e., hypoalbuminemia) can occur in patients with depression who develop drug resistance [15].

While more investigations are needed into the mechanism underlying the association between albumin level and depression, several possible explanations are as follows: Depression is characterized by a chronic low-grade inflammatory response, immune response, and oxidative and nitrosative stress with microprogrammed expression [28, 29]. Its etiology may be related to excess free radicals, which leads to oxidative stress and causes oxidative damage associated with neurodegeneration and various psychiatric disorders [13, 30]. Several studies have found oxidative stress to be involved in the pathogenesis of depression. Additionally, antioxidant and antioxidant enzyme production is reduced in depressive disorders, which leads to increased mitochondrial disease and dysfunction risk [31, 32], further suggesting that oxidative stress plays an important role in the development of depression. Meanwhile, serum albumin has free-radical scavenging properties [33] and strong associations with oxidative stress and antioxidant capacity [34], and albumin has been identified as an important nonenzymatic antioxidant [12]. Serum albumin is the main extracellular molecule responsible for maintaining the plasma redox status, and reduced albumin may lead to oxidative stress dysregulation, while higher free-radical and oxidative damage levels are detectable in patients with depression [13]. In addition, albumin is the main protein synthesized in the liver and can be negatively affected by inflammation, reflecting systemic inflammatory and immune dysfunction [35]. Depression is known to be strongly associated with immune activation and increased expression levels of various inflammatory markers, and serum albumin is involved in the inflammatory system and decreases as inflammation increases; therefore, reduced albumin levels may affect emotion via mechanisms such as the acute phase response versus immune response [35]. The symptomatology of depression has also been linked to various metabolites and molecules (e.g., fatty acids, magnesium ions, and thyroid hormones), omega-3 polyunsaturated fatty acid deficiency has been associated with depression and suicidal behaviors [36, 37], and patients with depression have reduced serum magnesium and zinc levels [38] as well as reduced synthesis and secretion of thyroid hormones [39]. Serum albumin can carry and transport these molecules and indirectly mediates the effects of other molecules on depression. Reduced albumin levels also reduces the availability of the essential amino acid tryptophan, which affects the production of 5-hydroxytryptamine, which is a depression-related neurotransmitter [19, 40].

This study had significant strengths that should be noted. Our findings can provide supportive evidence for clinical work involving adults with depressive symptoms, and low albumin levels should be adjusted to maintain the optimal status, especially in those with chronic diseases. Furthermore, this study was the first that we were aware of to investigate the association between depressive symptoms and album levels in adults. Our study, together with previous ones, have effectively illustrated that albumin may play a role as a clinical immunoinflammatory predictor in depressive symptoms and the depressive symptoms of other somatic diseases, providing some clues to the mechanisms of depression. Our findings also reveal a potential public health concern. We found that smoking status was significantly related to the association between albumin and depressive symptoms, with the negative association between albumin and depressive symptoms being stronger in smokers than in nonsmokers. This was consistent with a recent study finding that smoking strengthened the inverse association between albumin and diseases, and that cigarette smoking was inversely associated with serum albumin concentration [41]. However, the absence of a significant association could have been due to the small number of participants in the smoking group of our study, and so further research is needed to confirm this finding. It was noted that these existing disparities may affect the health of other persistent smokers.

Our study also had several inevitable limitations. Firstly, diagnosis of depression was established on self-rate scale of PHQ-9 in this study, lacking clinician-based diagnosis of depression, therefore we used the item of depressive symptoms objectively. Second, although the study sample was sufficiently large, causal associations between depressive symptoms and albumin levels could not be estimated due to its cross-sectional design, these data indicate the need for future studies involving larger samples and longer follow-ups in order to fully determine the role of albumin in depressive symptoms and investigate the durability of the associations identified in this study. Third, these findings might not be directly extrapolatable to other racial groups and regions and may only be mostly applicable to the US. Notwithstanding these limitations, this was a valuable population-based study focusing on the association between depressive symptoms and albumin levels in adults.

## Conclusions

Higher albumin concentration was associated with a lower risk of depressive symptoms in individuals in the general population, even after adjusting for case complexities. This finding indicates that albumin level may be an independent but auxiliary predictor of depression development.

## Electronic supplementary material

Below is the link to the electronic supplementary material.

Supplementary Material 1

## Data Availability

The datasets generated and analyzed for the current study are available in the NHANES repository. These data can be accessed using the following link: https://wwwn.cdc.gov/nchs/nhanes/Default.aspx.
