# Supplementary material for: The association between serum albumin and depressive symptoms: a cross-sectional study of NHANES data during 2005–2018
Source: BMC Psychiatry. 2023 Jun 20;23:448. doi: 10.1186/s12888-023-04935-1 (PMC10283330; doi:10.1186/s12888-023-04935-1)
Supplement: Supplementary file 1 — Supplementary Material 1 [file 12888_2023_4935_MOESM1_ESM.docx]

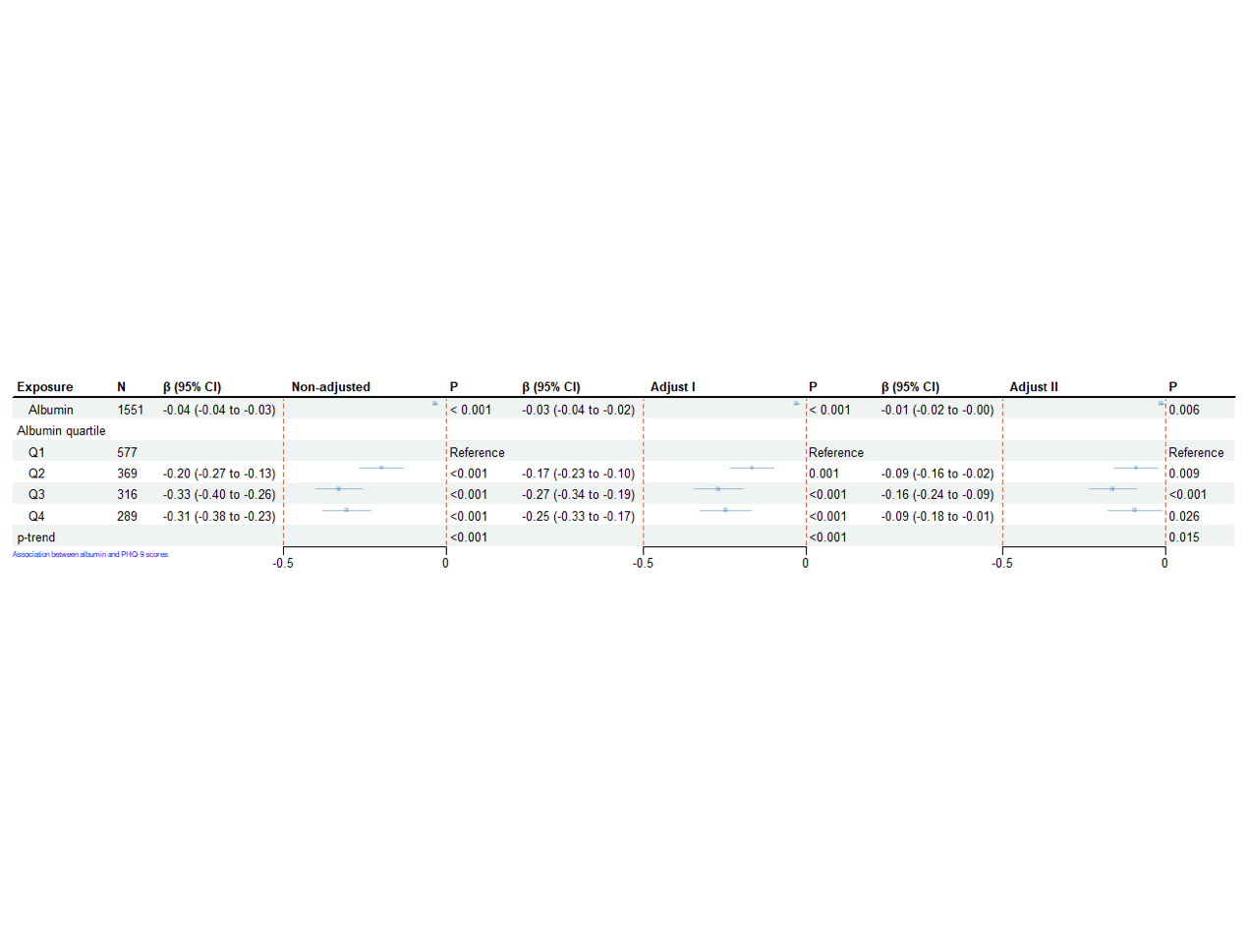


Supplementary Figure 1 Weighted association between albumin and decreased skewness of PHQ-9 scores in linear regression

Data are presented as β, 95% confidence intervals, and *p*-value.

Non-adjusted model adjusts for: none.

Adjust I model adjust for: age (years), gender, race.

Adjust II model adjust for: age (years), race, gender, education level, BMI status, drinking status, smoking status, congestive heart failure, coronary heart disease, liver condition, cancer or malignancy, diabetes, thyroid problem.
